# Pre-cut Filter Paper for Detecting Anti-Japanese Encephalitis Virus IgM from Dried Cerebrospinal Fluid Spots

**DOI:** 10.1371/journal.pntd.0004516

**Published:** 2016-03-17

**Authors:** Tehmina Bharucha, Anisone Chanthongthip, Soumphou Phuangpanom, Ooyanong Phonemixay, Onanong Sengvilaipaseuth, Manivanh Vongsouvath, Sue Lee, Paul N. Newton, Audrey Dubot-Pérès

**Affiliations:** 1 London School of Hygiene and Tropical Medicine, London, United Kingdom; 2 Lao-Oxford-Mahosot Hospital-Wellcome Trust Research Unit (LOMWRU), Microbiology Laboratory, Mahosot Hospital, Vientiane, Lao PDR; 3 Centre for Tropical Medicine and Global Health, Nuffield Department of Clinical Medicine, University of Oxford, Churchill Hospital, Oxford, United Kingdom; 4 Mahidol Oxford Tropical Medicine Research Unit, Faculty of Tropical Medicine, Mahidol University, Bangkok, Thailand; 5 UMR_D 190 "Emergence des Pathologies Virales" (Aix-Marseille Univ, IRD French Institute of Research for Development, EHESP French School of Public Health), Marseille, France; U.S. Naval Medical Research Unit No. 2, INDONESIA

## Abstract

**Background:**

The use of filter paper as a simple, inexpensive tool for storage and transportation of blood, ‘Dried Blood Spots’ or Guthrie cards, for diagnostic assays is well-established. In contrast, there are a paucity of diagnostic evaluations of dried cerebrospinal fluid (CSF) spots. These have potential applications in low-resource settings, such as Laos, where laboratory facilities for central nervous system (CNS) diagnostics are only available in Vientiane. In Laos, a major cause of CNS infection is Japanese encephalitis virus (JEV). We aimed to develop a dried CSF spot protocol and to evaluate its diagnostic performance using the World Health Organisation recommended anti-JEV IgM antibody capture enzyme-linked immunosorbent assay (JEV MAC-ELISA).

**Methodology and Principal Findings:**

Sample volumes, spotting techniques and filter paper type were evaluated using a CSF-substitute of anti-JEV IgM positive serum diluted in Phosphate Buffer Solution (PBS) to end-limits of detection by JEV MAC-ELISA. A conventional protocol, involving eluting one paper punch in 200μl PBS, did not detect the end-dilution, nor did multiple punches utilising diverse spotting techniques. However, pre-cut filter paper enabled saturation with five times the volume of CSF-substitute, sufficiently improving sensitivity to detect the end-dilution. The diagnostic accuracy of this optimised protocol was compared with routine, neat CSF in a pilot, retrospective study of JEV MAC-ELISA on consecutive CSF samples, collected 2009–15, from three Lao hospitals. In comparison to neat CSF, 132 CSF samples stored as dried CSF spots for one month at 25–30°C showed 81.6% (65.7–92.3 95%CI) positive agreement, 96.8% (91.0–99.3 95%CI) negative agreement, with a kappa coefficient of 0.81 (0.70–0.92 95%CI).

**Conclusions/Significance:**

The novel design of pre-cut filter paper saturated with CSF could provide a useful tool for JEV diagnostics in settings with limited laboratory access. It has the potential to improve national JEV surveillance and inform vaccination policies. The saturation of filter paper has potential use in the wider context of pathogen detection, including dried spots for detecting other analytes in CSF, and other body fluids.

## Author Summary

Japanese encephalitis virus infection is widespread in Asia, and primarily affects children in poor, rural areas. The virus spreads to the brain and spinal cord resulting in significant death and disability. Diagnosis requires testing for the immune response ‘antibody’ specific to Japanese encephalitis virus in the cerebrospinal fluid that surrounds the brain and spinal cord. However, in most areas where the infection occurs there are no laboratory facilities. In the absence of laboratory diagnosis, we have poor knowledge of the extent of the problem in these areas. Dried blood spots on filter paper have been used widely for many years as simple, cost-effective tools for transporting samples for testing. However, there have been few attempts at using dried cerebrospinal fluid spots. We developed a technique for using dried spots to store and test cerebrospinal fluid for antibodies to Japanese encephalitis virus. We compared the results of dried spots with routine neat cerebrospinal fluid in 132 patient samples, and demonstrated excellent agreement between the two tests. This novel method for saturating dried cerebrospinal fluid spots has the potential to enhance our knowledge of Japanese encephalitis virus epidemiology, and inform health policies where they are most needed. It could also be transferred for use in diagnosing other infectious diseases, including using other body fluid samples.

## Introduction

The last few decades have seen a substantial growth of novel and complex diagnostic tests [1]. This has not been accompanied by the same development in global laboratory infrastructure, a fundamental component of any effective healthcare system [2]–[4]. In settings with poor access to laboratories, the use of dried blood spots on filter paper (DBS) is now a well-established diagnostic tool for storing and transporting blood [5]–[9]. DBS obviates the need for a cold chain. The technique is also simple, economical and requires smaller sample volumes.

Strikingly, there is a paucity of data on use of dried spots of other body fluids [5]. There are only three publications evaluating the use of dried cerebrospinal fluid (CSF) spots (DCS) in diagnosing infectious diseases [10]–[12]. This may in part be due to the significant level of technical expertise required to perform a lumbar puncture (LP). CSF is more difficult to obtain, available in smaller volumes, often with lower concentrations of analyte, and may have lower sensitivity of corresponding diagnostic assays than for blood. Furthermore, approvals and practicalities of studies involving LPs are more challenging. It has been acknowledged that research involving CNS infections has been a neglected field [13]. However, it is also possible that more DCS methods have been tried, proved futile and were not published.

Amongst CNS infections, Japanese encephalitis virus (JEV) is recognised as the most common causative pathogen in Asia. In the 24 endemic countries it is suggested that JEV causes 67,900 cases, and 20,000 deaths per year [14], [15]. In the Lao PDR (Laos), there is evidence to suggest that JEV is a major cause of CNS infection [16], [17]. However, the wider epidemiology of JEV outside Vientiane is poorly documented due to the lack of laboratory facilities in the provinces. The diagnosis of JEV relies on laboratory facilities to test for anti-JEV IgM in the blood and/or CSF, with significantly increased proportion of false positive results if relying on blood alone [17]. Improved JEV laboratory networks and infrastructure have developed since 2008, with a global central laboratory in Japan, regional centres for the Western Pacific in China and Korea and respective national centres [18], [19]. The central laboratory performs the reference standard test, plaque reduction neutralisation assay (PRNT)[18]–[22]. However the mainstay of routine diagnosis involves commercial kits performing anti-JEV IgM antibody capture enzyme-linked immunosorbent assay (JEV MAC-ELISA).

The use of DCS in the diagnosis of JEV by JEV MAC-ELISA would have potential application in low-resource settings, for diagnostics, wider epidemiological studies on the aetiology of CNS infections and impact of vaccination. We aimed to optimise a DCS protocol for the detection of anti-JEV IgM using JEV MAC-ELISA and evaluate its performance in a pilot study of consecutive patient samples as compared to routine neat CSF.

## Methods

### Patient samples

**Fig 1:**
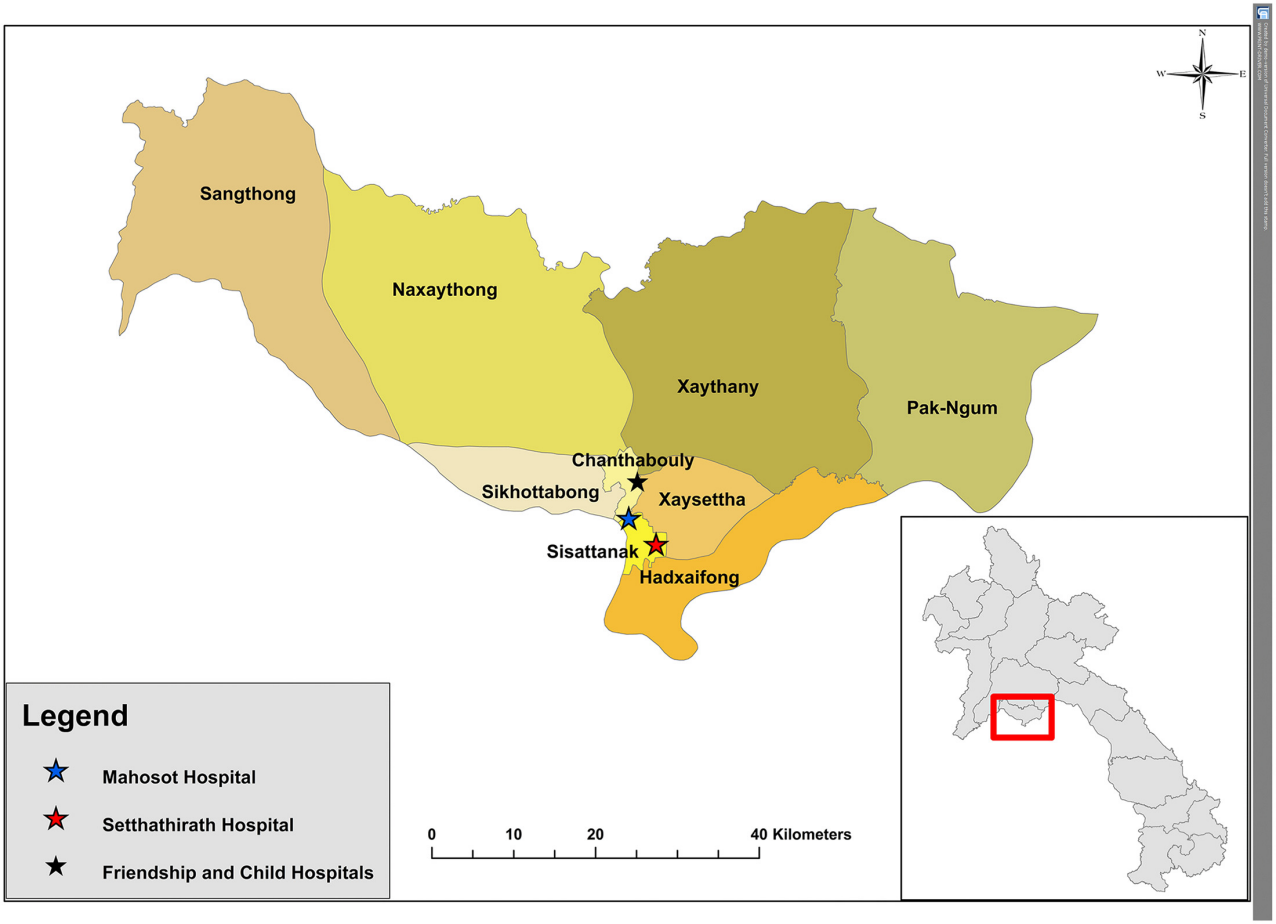
Study sites: Geographical distribution of four hospitals involved in patient recruitment, in Vientiane (colour map), and with respect to the Lao PDR (grey map).

Patient CSF and serum samples were collected at three hospitals in Vientiane, the Friendship, Children and Setthathirat Hospitals, from 2009 to 2015 (Fig 1). CSF was collected by LP from patients with suspected CNS infection, and without contraindications to LP, according to the judgment of the responsible physician. Written consent was obtained from the patient, parent and/or guardian. Samples were sent to Mahosot Hospital and stored at -80°C.

### Ethical considerations

The study was part of a study on the causes of CNS infections in Laos. Ethical clearance was granted by the Ethical Review Committee of the Faculty of Medical Sciences, National University of Lao, and the Oxford University Tropical Ethics Research Committee, Oxford, UK. Diagnostic testing was performed on anonymised and frozen CSF samples. LPs were performed as part of a routine diagnostic service if verbal (2003–2006) or written (2006–2011) consent was given by patients or their parents/guardian. Consent for all patients was documented on a form that had been approved by the ethics committee. The lead investigator has been trained in Good Clinical Practice, and has completed Human Tissues Training.

### Anti-JEV IgM ELISA (JEV MAC-ELISA)

The WHO recommended commercial JEV MAC-ELISA assay is the Inbios JE Detect (Washington, USA) [19]. This measures the Optical Density (OD) of each sample with JEV Recombinant Antigen, JERA, compared to Normal Control Antigen (NCA) to adjust for background nonspecific reactivity. 100μl of diluted sample (1/10 dilution for CSF as recommended by WHO) are used for a single test, 50μl in the JERA well and 50μl in the NCA well. The result is an Immune Status Ratio, ISR, (JERA OD/ NCA OD) with qualitative interpretation, according to manufacturer’s instructions, as positive if >6.0, equivocal if 4.0–6.0, or negative if <4.0. Equivocal results were classed as negative as per standard protocol in research optimising diagnostic tests [23], [24].

### Filter paper

Two types of filter paper were chosen, and utilised in all experiments. The 903 Protein Saver Card (903) (Whatman, GE Healthcare Life Sciences, UK) has been used as DBS for neonatal screening since 1963 [8]. It is made of highly purified cellulose cotton paper and is one of two FDA approved cards for DBS [5]. They have been used in the diagnosis of multiple diseases, involving elution and corresponding ELISA or PCR, including the detection of specific antibodies in CSF [25]. The 3MM Chr Blotting paper (3MM) (Whatman, GE Healthcare Life Sciences, UK) is available as large sheets, is less expensive and more suitable for broader application in a resource limited setting. Despite being produced as a blotting paper rather than a collection paper, 3MM has been used reliably in serology testing of DBS [26], [27].

### Optimisation experiments

#### CSF-substitute

CSF-substitutes were prepared to compare the end-limit of detection of the DCS method to the routine method from neat sample, and because of the limited CSF volumes available. A fluid was needed with similar consistency to CSF, and low IgM titre. Two anti-JEV IgM positive serum samples (A and B respectively) were diluted in PBS to the limit of detection-the highest dilution found positive by JEV MAC-ELISA. This is referred to here as the end-limit dilution, and was 1:1600 and 1:3200 for samples A and B, respectively. It was expected that there would be some loss in sensitivity when testing from dried spots so two additional dilutions, more concentrated, were also prepared: x2 (1:800 and 1:1600 for A and B respectively) and x4 end-limit dilutions (1:400 and 1:800 for A and B respectively). All the CSF-substitutes, three dilutions (end-limit, x2 end-limit and x4 end-limit) from the two sera A and B, were used in subsequent experiments. When directly used for ELISA testing, not spotted on paper, it is referred as neat CSF-substitute.

#### DCS preparation and elution

The DCS protocol was adjusted to compare sample volumes, spotting techniques and filter paper. The general protocol is described here, with the variables elaborated below. The CSF-substitutes were spotted on 3MM and 903 filter paper, with the centre of the spot marked as an indent with a pin. These were air-dried for 4 hours and stored in individual zip-lock plastic bags with a teaspoon of silica granules and then stored overnight in an air-conditioned laboratory at 25–30°C. The elution process involved inserting punched or pre-cut filter paper in 1.5ml Eppendorf tubes, adding different volumes of PBS/ 0.05% Tween-20, agitating on a shaker for 1 hour at 180 revs/minute at room temperature, and refrigerating overnight at 4°C. The tubes were then centrifuged for 10mins at 3,000g at 20°C and 100μl undiluted supernatant was used to perform JEV MAC-ELISA, using the JEV Detect InBios kit. Neat CSF-substitute was tested, diluted 1/10, on the same plate as per the manufacturer’s instructions. Each plate was validated with kit controls, as well as a PBS spotted on filter paper as an additional negative control.

A conventional DBS protocol was initially utilised, as per the in-house protocol for DBS and studies involving a wide-variety of analytes using DBS [7], [9]. 100μl of CSF-substitute was spotted on filter paper, and eluted as an 8mm punch (skin-puncher, Stiefel, Maidenhead, UK) in 200μl of PBS/0.05% Tween-20. Decontamination of the puncher was performed by punching filter paper soaked in Virchon 1%, and then three times in filter paper soaked in sterile water.

A modified protocol was used to increase the volume of CSF-substitute eluted using eight and ten 6mm punches in the minimum volume of PBS/0.05% Tween-20, 325μl, to provide the 275μl required eluate for subsequent JEV MAC-ELISA and potentially additional anti-dengue IgM and dengue NS1 assays. Different sample volumes were evaluated using the modified protocol with ten 6mm punches: 1) a single 100μl spot as previously described, 2) respotting a 100μl spot after completely air-drying a single spot, and 3) Two separate spots, with the innermost 5 punches taken from each spot.

A pre-cut circle protocol was performed completely saturating a pre-cut filter paper circle. Increasing volumes of PBS were spotted on pre-cut filter paper placed on a 3x3cm clear plastic square. The largest volume of PBS the circle could hold without the fluid seeping off was defined as the saturating volume. This was 250μl spotted on a 1.49cm diameter circle, five times the volume of CSF-substitute per surface area when spotted on a large area of paper. This entire circle was eluted in 200μl PBS/0.05%Tween-20.

#### Testing strategy

The two patient samples at each of three dilutions of CSF-substitutes were subjected to the various protocols. Experiments were performed as duplicates tested on the same JEV MAC-ELISA plate, and the entire protocol was repeated on another day. In total there were 4 to 6 replicates of each experiment. The JEV MAC-ELISA result is a binary categorical outcome ‘positive’ and ‘negative’, and results of experiments are summarised as percentage of replicates (4–6) positive for each sample and as a total (62–65).

### Evaluation of the optimised protocol using patient samples

Retrospective, consecutive analysis was performed on available CSF from all patients tested for JEV between February 2009 and July 2015. CSF was spotted on 903 paper as per the optimised ‘pre-cut circle’ protocol detailed above, and stored at 25–30°C for 30 days as an approximation of the maximum time for the paper to reach the laboratory if this was performed in the field. DCS and neat CSF were compared on the same plate. Investigators were blinded to the previous neat CSF JEV ELISA results.

### Statistics

The sample size (n = 86) was calculated with values for sensitivity of Inbios JEV MAC-ELISA = 53%, expected positive agreement DCS with neat CSF = 75%, and expected negative agreement = 98%, programming formulae (as per the CDC report referenced by T. Lee) based on estimation of a kappa coefficient, into Microsoft Excel [23], [28]. Results are presented in 2x2 tables, analysed by reporting ‘positive percentage agreement’ = (positive by both DCS and neat CSF)/(positive by neat CSF), ‘negative percentage agreement’ = (negative by both DCS and neat CSF)/(negative by neat CSF), overall percentage agreement (positive or negative agreement by both DCS and neat CSF) and kappa coefficient with respective 95% confidence intervals calculated in STATA 13.1 (College Station, Tx) [29]. The analysis acknowledges the fact that the comparison was not made with the reference standard, PRNT, that is only performed in a few reference laboratories. Further, the samples tested were paired, being taken from the same patient sample.

## Results

### Optimisation experiments

The conventional DBS protocol applied to DCS did not produce any positive results at the end-limit dilutions (Table 1A displays results for 903, for 3MM see S1 Table). The modified protocol with increased number of punches of the eluent, also did not produce any positive results at the end-limit dilutions (Table 1B and S1 Table). Experiments were performed using CSF-substitute at x2 and x4 end-limit dilutions, to identify the limit of detection of the modified protocol. At the lowest dilution, x4 end-limit dilution, 25% of replicates were positive using a single spot. The same dilution was used with a respotting technique and produced 75% positive replicates. A third technique using the same dilution spotted as two spots, with half the punches taken from the centre of each spot, was positive in 58% and 25% % of replicates, for 903 and 3MM, respectively. The only difference between the filter paper types was more frequent detection (58 vs 25%) using two spots on 903 versus 3MM.

**Table 1:** JEV MAC-ELISA results from DCS using CSF-substitutes, 903 filter paper and various protocols for sample spotting.

							Number of samples positive (%) by Inbios JE Detect								
							Total (SA+ SB), CSF-substitute A (SA) and CSF-substitute B (SB) at three dilutions								
Filter paper			Sample spotting	Punches/ Cutting			x4 End-limit dilution			x2 End-limit dilution			End-limit dilution		
**A) Conventional DBS Protocol**													**Total (n = 8)**	SA (n = 4)	SB (n = 4)
903	Single 100μl				1 (8mm)								**0 (0%)**	0 (0%)	0 (0%)
**B) Modified Protocol**							**Total (n = 12)**	SA (n = 6)	SB (n = 6)	**Total (n = 12)**	SA (n = 6)	SB (n = 6)	**Total (n = 12)**	SA (n = 6)	SB (n = 6)
903	Single 100μl				10 (6mm)		**3 (25%)**	2 (33%)	1 (17%)	**0 (0%)**	0 (0%)	0 (0%)	**0 (0%)**	0 (0%)	0 (0%)
903	Respot 100μl				10 (6mm)		**9 (75%)**	6 (100%)	3 (50%)	**2 (17%)**	2 (33%)	0	**0 (0%)**	0 (0%)	0 (0%)
903	Two 100μl				10 (6mm)		**7 (58%)**	6 (100%)	1 (17%)	**0 (0%)**	0 (0%)	0 (0%)	**0 (0%)**	0 (0%)	0 (0%)
**C) Pre-cut circle Protocol**													**Total (n = 8)**	SA (n = 4)	SB (n = 4)
903		Single 250μl				14.9mm							**8 (100%)**	4 (100%)	4 (100%)

DCS: Dried CSF Spot; DBS: Dried Blood Spot; Inbios JE Detect: WHO recommended commercial JEV MAC-ELISA for Anti-JEV IgM detection. CSF-substitutes SA and SB refer to the sera A and B that were used for their preparation. Modified Protocol: 3 sample volume techniques (Single/Respot/Double), 2 types of filter paper, 3MM/903, and 2 types of punches. End-limit dilution: highest dilution of serum found positive by Inbios JE detect, corresponding to the limit of detection of the kit when tested from neat CSF-substitute. For SA: End-limit dilution = 1/1600, x2 End-limit dilution = 1/800, x4 End-limit dilution = 1/400. For SB: End-limit dilution = 1/3200, x2 End-limit dilution = 1/1600, x4 End-limit dilution = 1/800.

The final protocol evaluated a pre-cut circle of filter paper saturated with the maximal volume of CSF. A 1.49cm diameter pre-cut circle could be loaded with 250μl CSF-substitute, and this permitted detection of anti-JEV IgM in 100% of replicates (Table 1C and S1 Table). In comparison, spotting 100μl on to a filter paper filled an approximate 2.1cm diameter circle for CSF and 1.3cm diameter sheet for blood [30]. Limitations of serum volume and JEV MAC-ELISA precluded testing all protocols at x2 and x4 end-limit dilutions. The pre-cut circle protocol with 903 filter paper was ultimately performed with 903 rather than 3MM filter paper in the optimised protocol as it showed marginally better results and was easier to handle.

### Evaluation utilising the optimised protocol

CSF was received and tested for JEV using Inbios JEV MAC-ELISA from 248 patients admitted from February 2009 to July 2015. 132 patients had sufficient frozen CSF volumes, 10μl for neat CSF ELISA testing and 250μl to make the dried CSF spot following pre-cut circle protocol. After 30 days at room temperature (25–30°C) the DCS and corresponding neat CSF were tested by Inbios JEV MAC-ELISA. The positive agreement for dried CSF spots compared to neat CSF was 81.6% (65.7–92.3 95%CI), negative agreement 96.8% (91.0–99.3 95%CI), overall agreement 92.4% and kappa coefficient 0.81 (0.70–0.92 95%CI), Table 2. Thirty-eight neat CSF samples (29%) and 34 filter paper samples (26%) tested positive for JEV.

**Table 2:** Anti-JEV IgM ELISA (JEV MAC-ELISA) results of consecutive patient samples comparing Dried Cerebrospinal Fluid Spots (DCS) on filter paper using the pre-cut protocol vs routine neat Cerebrospinal Fluid.

	Neat CSF		
	Positive	Negative	Total
**Filter paper**			
** Positive**	31	3	34
** Negative**	7	91	98
** Total**	38	94	132

## Discussion

There are still considerable global deficits in laboratory capacity for diagnosing CNS infections [2], [3]. This is seen in Laos, where laboratory diagnosis of JEV is confined to the capital, Vientiane, and elsewhere in rural Asia. DCS has the potential to provide a simple tool for improving clinical diagnostics and our understanding of the epidemiology of CNS infections. This study developed a robust protocol using DCS for the diagnosis of JEV by JEV MAC-ELISA, with excellent agreement demonstrated in a pilot study of patient samples.

The use of DBS for storing, transporting and detecting anti-JEV IgM and anti-dengue IgM have already been well-documented in previous studies, and this was not repeated here. [21], [31]–[34]

The experiments performed to optimise the DCS protocol highlighted the key difference between blood and CSF, with poor performance of the conventional DBS and modified protocols likely due to low IgM concentration in the final eluate, IgM concentrations are lower in CSF than in blood [35]. This was corroborated by the improved sensitivity seen by increasing the serum concentration in the modified DCS protocol to x4 end-limit dilution of the neat CSF-substitute. This problem is compounded by the lower viscosity of CSF, leading to a wider distribution on filter paper [30]. There was no evidence to suggest a difference in the performance of the two types of filter paper, 903 and 3MM. Respotting, aiming to increase the analyte concentration, did not improve sensitivity. There is literature to suggest that analyte concentrates in the centre of a DBS [36]–[38]. However taking half (4 or 5) the punches from the centre of two spots did not demonstrate higher sensitivity.

In contrast, the ‘pre-cut circle’ protocol worked very well. Saturation of filter paper could be achieved by cutting beforehand and then carefully loading with the maximum CSF volume the paper could hold. The volume of CSF that could be added to the pre-cut circle was five times that of a single CSF sample spotted on a sheet of filter paper. Prior cutting has been utilised in DBS [39], [40]. However, while the term ‘saturation’ is frequently used in dried spot protocols, this is not true saturation, by which we mean loading of fluid to the maximal amount that the filter paper will possibly absorb before it leaks off the filter paper. The use of this saturation technique has important implications in DBS in diagnostics for diseases that have low concentrations of analyte, dried spots involving other body fluids, as well as other fields of research. Advantages of the pre-cut circle protocol include greater sampling efficiency, as it prevents loss of the analyte not punched from the filter paper. It is simpler, quicker, more reliable and reduces concerns regarding cross-contamination.

The retrospective study of 132 consecutive patients recruited from three hospitals demonstrated a high level of agreement between the optimised ‘pre-cut’ circle protocol and neat CSF, with positive agreement of 81.6% (65.7–92.3 95%CI), negative agreement of 96.8% (91.0–99.3 95%CI) and kappa coefficient 0.81 (0.70–0.92 95%CI). It is possible that kappa agreement overestimates the effect size, as the comparison involved paired samples, tested using the same diagnostic assay. Further, kappa agreement has been criticised as being dependent on disease prevalence, with a low prevalence seen in this study risking a false negative result [41]. However, it is the industry standard [28], [29], [42]. P values for the kappa statistic are not presented as these are considered misleading [42]–[44].

10 of 132 (8%) CSF samples had discordant ELISA results between neat CSF and pre-cut saturated paper samples. Refreezing samples may have affected results. Unfortunately there was insufficient sample volumes to investigate this by the reference standard, PRNT.

Cross-reactivity with JEV MAC-ELISA occurs with other Flavivirus antibodies, and in this region, testing for dengue is crucial. We had insufficient sample volumes to test for dengue antibodies as well as JEV antibodies in the DCS. A larger study is needed to evaluate the differential diagnosis of JEV from other flaviruses, performing the protocol in ‘full’ with 500μl CSF, on a 2.1cm diameter circle, eluting with 400μl PBS/0.05% Tween-20.

Rules of thumb for the evaluation of any diagnostic test are the ‘ASSURED’ criteria, i.e. whether it is Affordable, Sensitive, Specific, User-friendly, Rapid and robust, Equipment-free and Deliverable to end-users [45]. The currently used WHO recommended standard test for JEV is the commercial Inbios JEV MAC-ELISA, CSF usually diluted 1:10 with a sensitivity under field conditions suggested to be 53% [23]. However, it is not accessible for the large populations at risk of JEV living in rural Asia. The DCS ‘pre-cut and saturated’ protocol potentially provides a simple, economical and accessible tool to store and transport CSF. The requirements for the technique are minimal, as PBS/ 0.05% Tween-20 and filter paper are easily available and inexpensive. Further work is crucial to evaluate the technique in larger cohorts, preparing dried CSF spots in multiple centres under field conditions, transporting the spots at the high ambient temperatures experienced in Asia, storing for longer periods (months to years), and testing paired with neat CSF. Ideally this could be performed as a diagnostic accuracy study, comparing both serum and CSF anti-JEV IgM ELISA results with PRNT, and with testing for anti-dengue IgM and Dengue NS1 antigen by JEV MAC-ELISA. It is notable that DCS may improve access to diagnostics in areas with poor laboratory facilities but does not reduce the time for diagnostic processing or ‘turnaround time’, and this is still a pertinent gap in this field. Permitting the collection, storage, and shipment of samples without the need for cold chain, this protocol could undoubtedly aid JEV surveillance by collecting data from rural hospitals with staff able to perform LPs that were not accessible before.

This novel design has significant implications for use in the wider context of pathogen detection, including dried spots methods for other analytes in CSF, and other body fluids. There are no facilities for systematic, population based surveillance of CNS infections in Laos outside the capital, as is the case in many parts of the world [46]. Even in countries where surveillance is performed, there are deficits in laboratory capacity, for diagnostics of JEV and other similar pathogens [3], [17], [23]. This affects both clinical management, outcome and epidemiological understanding. This is illustrated by the current epidemic of Zika virus, a related Flavivirus, with an unclear and poorly characterised association with neurological complications. There is an urgent need for simple and economical solutions to improve our understanding of the aetiology and wider epidemiology of CNS infections. With the introduction of nationwide JEV vaccination, it is likely that diagnosis by CSF will be even more important, and dried CSF spots could help understand the impact of vaccination.

## Supporting Information

S1 Table JEV MAC-ELISA results from DCS using CSF-substitutes, and various protocols for sample spotting, for filter papers 903 and 3MM. (DOCX)
