# Supplementary material for: Pre-cut Filter Paper for Detecting Anti-Japanese Encephalitis Virus IgM from Dried Cerebrospinal Fluid Spots
Source: PLoS Negl Trop Dis. 2016 Mar 17;10(3):e0004516. doi: 10.1371/journal.pntd.0004516 (PMC4795698; doi:10.1371/journal.pntd.0004516)
Supplement: S1 Table — (DOCX) [file pntd.0004516.s001.docx]

Supporting information

S1 Table: JEV MAC-ELISA results from DCS using CSF-substitutes, and various protocols for sample spotting for filter papers 903 and 3MM.

|  | | | | | **Number of samples positive (%) by Inbios JE Detect**  **Total (SA+ SB), CSF-substitute A (SA) and CSF substitute B (SB) a three dilutions** | | | | | | | | | | |
| --- | --- | --- | --- | --- | --- | --- | --- | --- | --- | --- | --- | --- | --- | --- | --- |
| **Filter paper** | | **Sample spotting** | | **Punches/cutting** | **x4 End-limit dilution** | | | **x2 End-limit dilution** | | | **End-limit dilution** | | | | |
| **A) Conventional DBS Protocol** | | | | |  | | |  | | | **Total (n=8)** | | SA (n=4) | | SB (n=4) |
| 3MM | Single 100µl | | 1 (8mm) | |  | | |  | | | **0 (0%)** | | 0 (0%) | | 0 (0%) |
| 903 | Single 100µl | | 1 (8mm) | |  | | |  | | | **0 (0%)** | | 0 (0%) | | 0 (0%) |
| **B) Modified Protocol** | | | | | | | | | | | | | | | |
| **Ten 6mm punches** | | |  | | **Total (n=12)** | SA (n=6) | SB (n=6) | **Total (n=12)** | SA (n=6) | SB (n=6) | **Total (n=12)** | | SA (n=6) | | SB (n=6) |
| 3MM | Single 100µl | | 10 (6mm) | | **3 (25%)** | 3 (50%) | 0 (0%) | **0 (0%)** | 0 (0%) | 0 (0%) | **0 (0%)** | | 0 (0%) | | 0 (0%) |
| 3MM | Respot 100µl | | 10 (6mm) | | **9 (75%)** | 6 (100%) | 3 (50%) | **2 (17%)** | 2 (33%) | 0 (0%) | **0 (0%)** | | 0 (0%) | | 0 (0%) |
| 3MM | Two 100µl | | 10 (6mm) | | **3 (25%)** | 3 (50%) | 0 (0%) | **0 (0%)** | 0 (0%) | 0 (0%) | **0 (0%)** | | 0 (0%) | | 0 (0%) |
| 903 | Single 100µl | | 10 (6mm) | | **3 (25%)** | 2 (33%) | 1 (17%) | **0 (0%)** | 0 (0%) | 0 (0%) | **0 (0%)** | | 0 (0%) | | 0 (0%) |
| 903 | Respot 100µl | | 10 (6mm) | | **9 (75%)** | 6 (100%) | 3 (50%) | **2 (17%)** | 2 (33%) | 0 (0%) | **0 (0%)** | | 0 (0%) | | 0 (0%) |
| 903 | Two 100µl | | 10 (6mm) | | **7 (58%)** | 6 (100%) | 1 (17%) | **0 (0%)** | 0 (0%) | 0 (0%) | **0 (0%)** | | 0 (0%) | | 0 (0%) |
| **Eight 6mm punches** | | |  | |  | | |  | | | **Total (n=8)** | | SA (n=4) | | SB (n=4) |
| 3MM | Single 100µl | | 8 (6mm) | |  | | |  | | | **0 (0%)** | | 0 (0%) | | 0 (0%) |
| 3MM | Respot 100µl | | 8 (6mm) | |  | | |  | | | **0 (0%)** | | 0 (0%) | | 0 (0%) |
| 3MM | Two 100µl | | 8 (6mm) | |  | | |  | | | **0 (0%)** | | 0 (0%) | | 0 (0%) |
| **C) Pre-cut circle Protocol** | | | | |  | | |  | | | **Total (n=8)** | SA (n=4) | | SB (n=4) | |
| 3MM | Single 250µl | | 14.9mm | |  | | |  | | | **8 (100%)** | 4 (100%) | | 4 (100%) | |
| 903 | Single 250µl | | 14.9mm | |  | | |  | | | **8 (100%)** | 4 (100%) | | 4 (100%) | |

DCS: Dried CSF Spot; DBS: Dried Blood Sopt; Inbios JE Detect: WHO recommended commercial JEV MAC-ELISA for Anti-JEV IgM detection. CSF-substitutes SA and SB refer to the sera A and B that were used for their preparation.Modified Protocol: 3 sample volume techniques (Single/Respot/Double), 2 types of filter paper, 3MM/903, and 2 types of punches. End-limit dilution: highest dilution of serum found positive by Inbios JE detect, corresponding to the limit of detection of the kit when tested from neat CSF-substitute. For SA: End-limit dilution= 1/1600, x2 End-limit dilution=1/800, x4 End-limit dilution=1/400. For SB: End-limit dilution= 1/3200, x2 End-limit dilution=1/1600, x4 End-limit dilution=1/800.
